# Effect of stress inoculation on reducing performance anxiety among physical education students

**DOI:** 10.3389/fspor.2026.1871990

**Published:** 2026-09-24

**Authors:** Rawan Abdul Mahdi Neyef Al-Saliti, Hamed Mohammed Ali Doum, Mohammad A. Tashtoush, Alaaeldin Ahmed Hamid, Sultan Mubarak Almughyirah, Abdelrahim Fathy Ismail

**Affiliations:** 1 Department of Psychological and Family Counseling, College of Educational Sciences, Ajloun National University, Ajloun, Jordan; 2 Department of Physical Education, College of Educational Sciences, Ajloun National University, Ajloun, Jordan; 3 Department of Educational Sciences, Ajloun University College, AL-Balqa Applied University, Salt, Jordan; 4 Department of Basic Sciences, AL-Huson University College, AL-Balqa Applied University, Salt, Jordan; 5 Faculty of Education and Arts, Sohar University, Sohar, Oman; 6 Department of Curriculum and Instruction, College of Education, King Faisal University, Al Ahsa, Saudi Arabia

**Keywords:** anxiety, cognitive behavioral therapy, performance anxiety, stress inoculation, students

## Abstract

**Objective:**

This study aimed to investigate the effect of stress inoculation on reducing performance anxiety among university students enrolled in Physical Education.

**Methodology:**

The study sample consisted of 30 s-year university students enrolled in the Physical Education program at Ajloun National University in Jordan. A quasi-experimental design was used to evaluate the effectiveness of the intervention program. Pre- and post-tests were administered to both the experimental and control groups, followed by a follow-up assessment. A performance anxiety scale, specifically developed for this study, was used for the assessments. The stress inoculation program was designed to provide students with cognitive and behavioral coping skills through three main stages: preparation and psychoeducation, acquisition and practice of coping skills, and application of these skills to performance-related situations.

**Results:**

The results showed that the designed program was effective in reducing performance anxiety among students in the experimental group compared with the control group. The results also indicated no statistically significant differences in the experimental group's performance anxiety scores between the post-test and follow-up assessments, suggesting that the reduction in anxiety was maintained during the follow-up period.

**Conclusion:**

The findings suggest that stress inoculation is an effective method for reducing performance anxiety among university students enrolled in Physical Education and may help them cope more effectively with performance-related pressures.

## Introduction

In the highly competitive world of sports, performance is a fundamental and indispensable element. It serves as a crucial criterion for judging an athlete's competence and progress (1–3). As competition intensifies and the spotlight shifts to achievement, performance anxiety emerges as a hidden adversary that accompanies athletes and can limit their ability to realize their full potential (1). A range of psychological factors intertwine with performance anxiety, most notably perceived training patterns. These training patterns shape behaviors and experiences within the sporting environment. Therefore, they may have a positive or negative impact on his performance level depending on the nature of this interaction (4), whereas anxiety constitutes an internal state that affects the athlete's cognitive and emotional responses (5–7). Performance anxiety is a common psychological phenomenon in sports. It appears in athletes at all levels, especially in situations requiring evaluation or direct competition (8, 9). This anxiety manifests as a state of tension and excessive preoccupation linked to the fear of failure or negative evaluation. It directly affects concentration, decision-making, and the ability to execute skills efficiently (10). Therefore, this concept has received considerable attention in the sports psychology literature due to its close connection to performance quality and achievement (11).

Among university students majoring in physical education, performance anxiety may emerge from the specific demands of practical physical education courses and the evaluative nature of motor performance. Students are frequently required to demonstrate physical and motor skills in front of university instructors and peers, which may increase concerns about making mistakes, receiving negative evaluation, or failing to meet expected performance standards. Research involving university physical education students has demonstrated that cognitive and somatic anxiety are important dimensions of performance-related anxiety and are associated with emotional and psychological factors relevant to sporting performance (12). Such evaluative situations may contribute to somatic anxiety through physiological arousal, including increased heart rate, muscle tension, sweating, and physical discomfort. At the cognitive level, students may experience worry about failure, negative expectations, and excessive concern about their performance, which may interfere with concentration and task execution. Evidence from sport and performance research indicates that cognitive and somatic anxiety are meaningful components of performance anxiety and may influence performance under pressure (9, 13). In addition, anxiety-related self-monitoring and concern about performance may interfere with attentional control and increase distractibility during practical activities. The difficulty and complexity of motor tasks may further contribute to anxiety when students perceive the required skills as exceeding their current abilities or when they have limited confidence in their capacity to perform them successfully. Moreover, the instructional and evaluative environment, including the nature of feedback provided by university instructors, opportunities for practice, perceived evaluation, and the social context of performing in front of peers, may shape students' emotional and cognitive responses (14, 15). Therefore, performance anxiety among university physical education students may result from the interaction of physiological arousal, performance-related cognitions, attentional difficulties, perceived task difficulty, self-confidence, and characteristics of the instructional and evaluative environment (9, 12).

Studies have focused on interpreting performance anxiety through its physiological and psychological dimensions, such as arousal levels, stress, and cognitive interference (5, 16, 17). These approaches have contributed to building the theoretical foundations for understanding the nature of anxiety and its effects. With the development of scientific research, performance anxiety is no longer viewed as a one-dimensional phenomenon. Rather, it is now understood within a multidimensional framework of cognitive, emotional, and behavioral aspects, such as emotion regulation, self-confidence levels, and the coping strategies athletes employ to control stress.

Therefore, performance anxiety is considered a negative factor affecting athletes' performance levels. It causes psychological stress, impaired concentration, and decreased efficiency during competitive situations (9). Hence, the need arose to design specialized counseling programs aimed at reducing levels of this anxiety and helping athletes control their emotional responses. Stress inoculation is a modern and effective method in sports psychology counseling. It aims to equip athletes with psychological skills that enable them to cope with competitive pressures and minimize their negative effects. This, in turn, contributes to improved psychological adaptation and enhanced performance (17). In this context, coping skills reflect the pivotal role of cognitive processes in determining an individual's response to different situations, especially under high levels of stress and anxiety. These skills maintain and improve performance levels during competition (18). Stress inoculation is considered one of the most prominent applied methods in the sports field. This approach aims to train athletes to develop effective coping skills and manage competitive pressures in a structured and gradual manner (19). It is based on a progressive principle of training coping skills first in low-stress situations, then transferring them to more stressful ones. This method enhances the athlete's ability to regulate their emotional and cognitive responses during competition (19, 20). Stress inoculation consists of three overlapping phases: preparation or education, skill acquisition, and application or transfer. The transition between these phases is integrated to ensure the consolidation of acquired skills (19). Practical applications of this approach indicate its effectiveness in helping athletes who struggle to perform under pressure. Training them to use effective coping strategies in situations to simulate real-world competition would reduce their anxiety levels and improve their performance.

A literature review indicates a growing interest in performance anxiety among physical education students. However, most studies have focused on measuring anxiety levels or correlating them with other variables. At the same time, the use of stress desensitization programs as a direct therapeutic approach to reducing performance anxiety remains relatively limited. In addition, studies addressing this type of program have often been conducted within general anxiety frameworks, lacking sufficient specificity for performance anxiety among physical education students. Moreover, the experimental application of stress desensitization programs to this group remains limited, despite the nature of performance-related stressors among them. Therefore, the current study aims to bridge this gap by investigating the effectiveness of stress desensitization programs in reducing performance anxiety among physical education students.

This study aims to test the following two hypotheses:

Hypothesis 1: There are no statistically significant differences at (*α*=0.05) between the mean scores of the experimental group and the control group on the athletes' performance anxiety scale attributable to the training program.
Hypothesis 2: There are no statistically significant differences at (*α*=0.05) between the mean scores of the experimental group in the post-test and follow-up on the athletes' performance anxiety scale attributable to the persistence of the training program's effect.

## Methods

The current study utilized a quasi-experimental design to investigate the effect of stress inoculation on reducing performance anxiety among physical education students. The design included two groups: an experimental group that participated in the stress inoculation-based intervention program, and a control group that did not receive this intervention during the implementation period. Pre-and post-tests were administered to both the experimental and control groups to measure performance anxiety levels before and after the intervention. To achieve the study's objectives, a performance anxiety scale was used to assess the performance anxiety levels of the physical education students. Students in the experimental group participated in stress inoculation sessions. Pre-and post-tests verified the effectiveness of the intervention program.

### Group equivalence: performance anxiety scale

To verify group equivalence, the Mann–Whitney U test was used to determine the significance of differences in pre-test scores of athletes on the performance anxiety scale in both the experimental and control groups. Table 1 illustrates these results.

**Table 1:** Results of the Mann–Whitney U test for determining the significance of differences in pre-test performance of the experimental and control groups on the performance anxiety scale.

Domains	Group	No.	Mean average	Mean total	Mean	Mann–Whitney U	z	Sig.
Somatic symptoms	Exp.	15	12.80	192.00	2.96	72.000	−1.701	0.089
	Con.	15	18.20	273.00	3.44			
Cognitive anxiety	Exp.	15	14.10	211.50	3.17	91.500	−0.877	0.380
	Con.	15	16.90	253.50	3.56			
Distractibility	Exp.	15	13.67	205.00	3.16	85.000	−1.148	0.251
	Con.	15	17.33	260.00	3.55			
Total	Exp.	15	12.70	190.50	3.10	70.500	−1.745	0.081
	Con.	15	18.30	274.50	3.52			

Table 1 shows that there are no statistically significant differences (a = 0.05) attributable to the group on the performance anxiety scale in all dimensions and the overall score. This result indicates the equivalence of the groups.

### Participants

A sample of 30 s-year students majoring in Physical Education at Ajloun National University in Jordan was selected using a performance anxiety scale. Students with high performance anxiety scores were deemed eligible to participate in the study, as this aligned with the intervention objectives. After obtaining informed consent from the participants, they were randomly assigned to two groups: an experimental group that underwent a stress inoculation program and a control group that received no intervention during the program's implementation. Each group consisted of 15 students. This randomization allowed the study to focus on physical education students who most needed intervention and enhanced the reliability of comparisons between the experimental and control groups, the sample selection procedure is clearly illustrated in the following flowchart (Figure 1).

**Figure 1:**
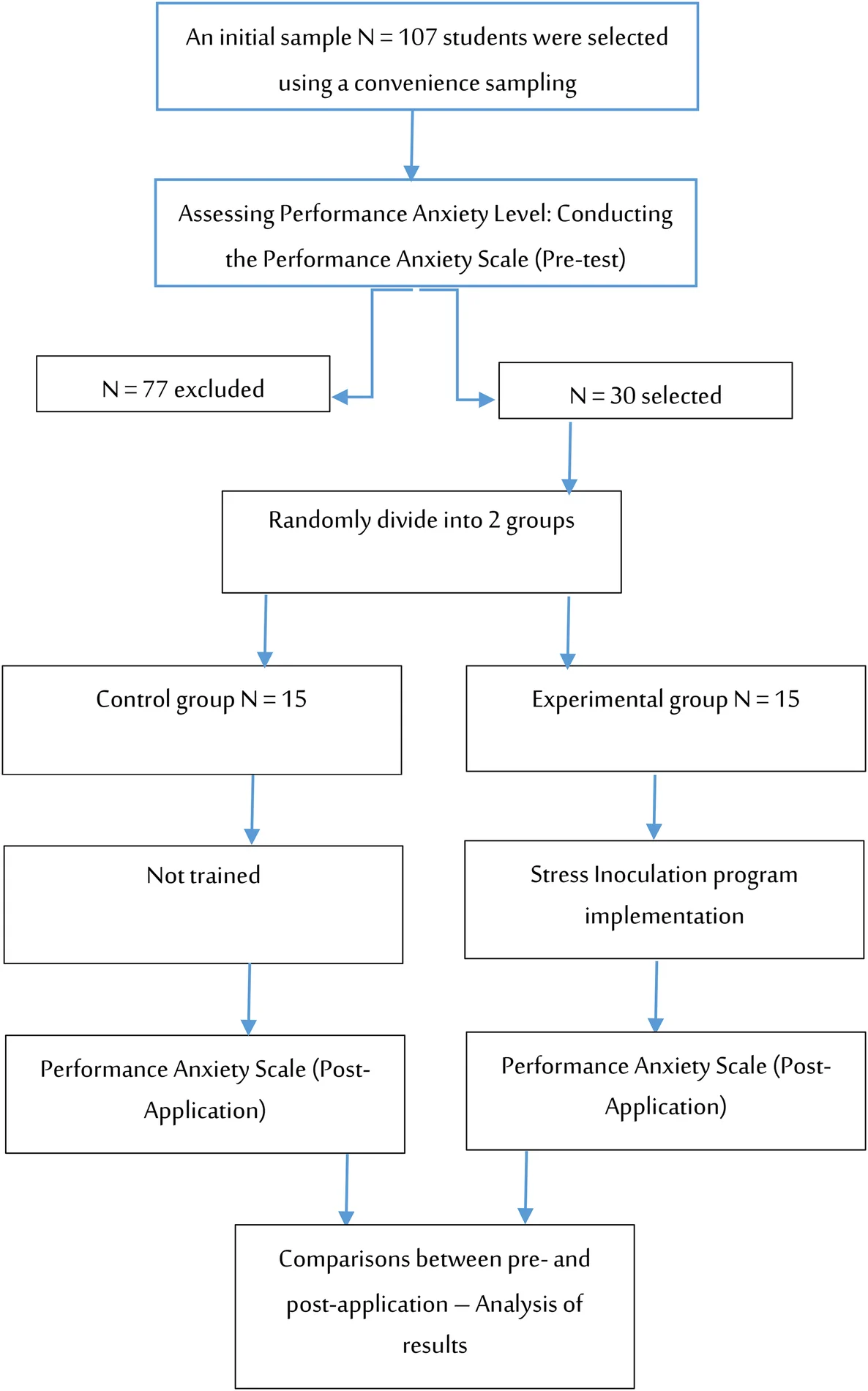
Sample selection procedures.

### Ethical considerations

The ethical considerations for this study included ensuring that participants were fully informed of the study's objectives, procedures, and potential risks. Participation was entirely voluntary, with all participants providing informed consent before joining. This study was approved by the Institutional Review Board of Ajloun National University, Jordan, under protocol number [IRB No. 4025/UN37.11/TU/2025], dated November 2025. Informed verbal consent was obtained from all participants, and all data were anonymized to protect participant confidentiality. The confidentiality and privacy of participant data were guaranteed, and no personally identifiable information was used in the analysis or scientific publication. Participants were informed of their right to withdraw from the study at any time without adverse consequences. The intervention program was designed to provide a safe and supportive psychological environment, minimizing any potential harm. The collected data were used solely for scientific research purposes and in accordance with established ethical standards for psychological research.

### Data collection

Performance anxiety scale: The performance anxiety scale used in the study by (21) is a psychological tool used to measure athletic performance anxiety in individuals. It is based on a cognitive-affective theory model that posits anxiety as composed of several interrelated dimensions. The scale consisted of 15 items distributed across three dimensions. The first dimension, Somatic Symptoms, included items 1–5 and refers to the physiological symptoms associated with anxiety, such as rapid heartbeat, muscle tension, and stomach upset. The second dimension, Cognitive Anxiety, included items 6–10 and reflects negative thoughts and mental preoccupation related to performance, such as fear of failure or anxiety about negative outcomes. The third dimension, Distractibility, included items 11–15 and expresses difficulty concentrating on the task or distractibility during athletic performance.

Validity and reliability of the original scale: The Sports Anxiety Scale (SAS-2) exhibits high levels of validity and reliability. Construct validity was confirmed using confirmatory factor analysis. The results affirmed the suitability of the three-dimensional model, which comprises somatic anxiety, cognitive anxiety (preoccupation), and distractibility. The fit indices showed good values (CFI = 0.95–0.97, NNFI = 0.95–0.96, RMSEA = 0.05–0.065), indicating the scale's construct quality. The scale also demonstrated high internal reliability, with Cronbach's alpha coefficients ranging from 0.73 to 0.86 for the subdimensions and 0.88 for the overall score. Furthermore, test-retest reliability was confirmed, with values ranging from 0.73 to 0.80, indicating the stability of the measurement over time. Therefore, the scale is a valid and reliable instrument for measuring multidimensional sports performance anxiety.

### Validity of the performance anxiety scale in the current study

To determine the construct validity of the performance anxiety scale, correlation coefficients were calculated for each item and the total score, for each item and its corresponding domain, and for the domains themselves and the total score. The scale was administered to a pilot sample of 30 participants, separate from the main study sample. The correlation coefficients between the items and the instrument as a whole ranged from 0.37 to 0.80, and between the items and the domains from 0.46 to 0.81, as shown in Table 2.

**Table 2:** Correlation coefficients between items, total score, and domains on the performance anxiety scale.

Item	Correlation coefficient (domain)	Correlation coefficient (scale)	Item	Correlation coefficient (domain)	Correlation coefficient (scale)	Item	Correlation coefficient (domain)	Correlation coefficient (scale)
1	0.58**	0.41*	6	0.76**	0.62**	11	0.81**	0.67**
2	0.74**	0.80**	7	0.70**	0.58**	12	0.53**	0.37*
3	0.46*	0.37*	8	0.53**	0.52**	13	0.68**	0.53**
4	0.55**	0.41*	9	0.77**	0.52**	14	0.69**	0.52**
5	0.59**	0.53**	10	0.75**	0.60**	15	0.79**	0.59**

* sig at (0.05).

** sig at (0.01).

It should be noted that all correlation coefficients were statistically significant and acceptable, therefore none of the items were removed.

The correlation coefficient between the domain and the total score, as well as the correlation coefficients between the domains themselves, were calculated. Table 3 illustrates the results.

**Table 3:** Correlation coefficients between domains and the total score of the performance anxiety scale.

Domain	Somatic symptoms	Cognitive anxiety	Distractibility	Performance anxiety scale
Somatic symptoms.	1.			
Cognitive anxiety.	*0.421.	1.		
Distractibility.	*0.446.	*0.390.	1.	
Performance anxiety scale.	**0.744.	**0.801.	**0.759.	1.

* sig at (0.05).

** sig at (0.01).

Table 3 shows that all correlation coefficients between the domains of the performance anxiety scale were positive and statistically significant at the 0.01 level. The correlation coefficients were high and statistically acceptable. Correlation coefficients exceeding 0.30 are considered significant for a valid correlation in educational and psychological studies. These results indicate a high degree of internal consistency between the scale's domains and the total score, and thus support the scale's construct validity.

### Reliability of the performance anxiety scale in the current study

To measure the reliability of performance anxiety, the test-retest method was applied. The scale was administered and then re-administered two weeks later to a control group of 30 participants outside the study sample. Pearson's correlation coefficient was then calculated between their scores on both occasions.

The reliability coefficient was also calculated using Cronbach's alpha for internal consistency. Table 4 shows the Cronbach's alpha for internal consistency, test-retest reliability for the domains, and the total score. These values were considered suitable for the purposes of this study.

**Table 4:** Cronbach's alpha for internal consistency, test-retest reliability for the domains, and total score for the performance anxiety scale.

Domain	Test-retest	Cronbach's alpha
Somatic symptoms	0.84	0.82
Cognitive anxiety	0.87	0.76
Distractibility	0.86	0.71
Performance anxiety scale	0.90	0.85

Table 4 shows that the study instrument has good levels of reliability. Internal consistency coefficients ranged from 0.71 to 0.85, and test-retest reliability coefficients ranged from 0.84 to 0.90. This result indicates a high level of reliability.

### Data analysis

Appropriate non-parametric statistical methods were used to examine the effect of the training program on reducing performance anxiety among physical education students. Also, they verified the sustainability of its effect over time. In addition, suitable statistical tests were used to determine the significance of differences between the experimental and control groups in the post-test and to compare the experimental group's performance between the post-test and follow-up tests. To test the first hypothesis, the means, ranks, and standard deviations of the physical education students' performance anxiety scores in the post-test were calculated according to the group variable (experimental and control). Moreover, the Mann–Whitney U test was applied to determine the significance of differences between the two groups. To test the second hypothesis, the means, ranks, and standard deviations of the experimental group's performance anxiety scores in the post-test and follow-up tests were calculated. The Wilcoxon signed-rank test was used to determine the significance of differences between the two applications.

### Intervention package: A counseling program based on stress inoculation therapy

A counseling program based on stress inoculation therapy was designed to reduce performance anxiety among physical education students. The design of the therapy program in this study was based on recent literature (20–24). The program was implemented with second-year students majoring in Physical Education at Ajloun National University who had high levels of performance anxiety. The intervention was conducted in the Psychological Counseling Laboratory, specifically in the Group Counseling Room, and was delivered by a faculty member with a PhD in Psychological Counseling, who was one of the researchers in the present study, is licensed by the Jordanian Ministry of Health to practice as a psychological counseling specialist, and is qualified and experienced in psychological counseling and in delivering structured counseling interventions.

The program provided a structured psychological intervention focused on developing cognitive and behavioral coping skills to help students deal with stressful sports situations more effectively. It included training in relaxation, cognitive restructuring, positive self-talk, visualization, and other stress-management techniques. The program also enhanced students' awareness of the relationship between thoughts, emotions, and performance. Particular emphasis was placed on modifying negative beliefs associated with fear of failure and concerns about being judged by others, and replacing them with more realistic and positive thoughts that foster self-confidence and support improved athletic performance.

The program consisted of 13 structured counseling sessions, conducted twice a week, with each session lasting approximately two hours (Table 5). The sessions followed a planned sequence of stress inoculation components, including preparation and identification of stressors, development and rehearsal of coping skills, and application of these skills in situations simulating the real sports environment, such as competitions and practical examinations. The sessions also emphasized managing physiological and emotional stress responses, reducing distractions, improving concentration, and restructuring negative cognitive and emotional experiences associated with performance anxiety. Through repeated practice and application, students were trained to use these coping skills when facing sports-related pressures, thereby strengthening their perceived competence and confidence and supporting more consistent and effective performance.

**Table 5:** Summary of program sessions.

Objectives	Session	No.
Getting to know each other and building a mentoring relationship between the mentor and the members, clarifying the nature of the program and its objectives, and discussing the members' expectations of the program.	Getting to know each other and building a mentoring relationship	1
Introducing members to the concept of sports performance anxiety and helping them recognize its various manifestations, such as cognitive anxiety, somatic anxiety, and attention deficit. This includes discussing the most common sports situations that trigger their anxiety and enhancing their understanding of the relationship between thoughts, feelings, and athletic performance.	Performance anxiety	2
This session aims to introduce members to the concept of stress inoculation as an effective guidance method for managing psychological stress, clarifying its objectives in the sports field, and its role in reducing performance anxiety and improving coping abilities. The session also seeks to help members understand the relationship between stress and performance anxiety, identify situations where this method can be applied, such as sports competitions and practical tests, and enhance members' motivation to commit to the program and apply its skills in their athletic lives.	Stress inoculation	3
This session aims to introduce members to cognitive coping skills as a key component in managing performance-related stress. The nature of these skills and their role in modifying negative thoughts associated with competition will be explained. The session also seeks to train members in cognitive coping strategies, such as reinterpreting stressful situations in a more positive light, and to model these strategies for them. In turn, it helps them adopt more balanced thinking patterns that contribute to reducing performance anxiety.	Acquiring cognitive coping skills	4
This session aims to help members learn adaptive, hands-on skills that can be used in stressful sports situations that impair their performance, such as during competitions or evaluations. They will also be trained to apply these skills practically through realistic role-playing scenarios, demonstrating how to use them effectively. This empowers members to cope with stress in a positive way that improves their performance and reduces their anxiety levels.	Acquiring, training, and modeling coping hands-on skills	5
This session aims to introduce members to stress-reduction strategies as immediate techniques to help reduce tension and anxiety in stressful situations, clarifying their purpose and importance in managing emotional responses during athletic performance. Members will also be trained to practice these strategies within the session, such as self-calming and focus, which will help them regain emotional balance and reduce performance anxiety.	Accommodation strategies	6
This session aims to introduce members to the concept of mental relaxation and its importance in reducing stress and improving focus during athletic performance by demonstrating its effect on reducing anxiety and promoting positive thinking. Members will also be trained to apply mental relaxation techniques within the session, such as positive visualization and mindfulness, thereby improving their ability to control their thoughts and emotions in competitive situations.	Mental relaxation	7
This session aims to introduce members to the concept of physical relaxation, clarifying the difference between it and mental relaxation, and explaining its role in reducing physical tension associated with performance anxiety, such as rapid heartbeat or muscle tension. Members will also be trained in applying physical relaxation techniques, helping them achieve balance between their physical and mental well-being, and thus improve their athletic performance.	physical relaxation	8
This session aims to help members identify their thought patterns, particularly negative thoughts related to athletic performance, such as fear of failure or anxiety about being judged by others. The session also seeks to clarify the role of these thoughts in increasing performance anxiety and to help members understand their nature and impact on their feelings and behavior during competition.	Identifying ideas	9
This session aims to train members in the skill of modifying irrational thoughts and replacing them with rational and positive ones, thereby reducing performance anxiety and improving self-confidence. Members are also guided on how to discuss and challenge their negative thoughts, replacing them with realistic ones that support effective performance in athletic situations.	Transforming irrational thoughts into rational ones	10
This session aims to integrate and apply the cognitive coping skills practiced in previous sessions through situations that simulate the actual sporting environment. It also seeks to enhance the participants' ability to use these skills automatically during performance. This contributes to positive changes in their cognitive structure, reduces performance anxiety, and improves their ability to cope with stress.	Applying cognitive coping skills	11
This session aims to review and discuss the cognitive and behavioral coping skills acquired during the program, and to enhance members' ability to integrate these skills into high-pressure athletic situations. These skills are modeled through positive visualization of competitive scenarios, allowing members to practice applying various strategies in a safe, realistic environment. The session also seeks to prepare members psychologically for the completion of the mentoring program and to foster a sense of competence in independently managing performance anxiety.	Applying all cognitive and behavioral coping skills	12
This session aims to evaluate the impact of the mentoring program by discussing with members the most significant benefits gained and the changes in their performance anxiety levels. It also addresses any negative aspects or difficulties encountered during implementation. The session includes a post-test to verify the program's effectiveness, reinforce members' motivation to continue applying the acquired skills in their athletic careers, and conclude the program on a positive note that supports their psychological independence.	Concluding session	13

## Results

Hypothesis 1: There are no statistically significant differences at (*α*=0.05) between the mean scores of the experimental group and the control group on the athletes' performance anxiety scale attributable to the training program.

To verify this hypothesis, the Mann–Whitney U test was used to determine the significance of differences in the athletes' scores on the performance anxiety scale in the post-test between the experimental and control groups. Table 6 shows the results.

**Table 6:** Results of the Mann–Whitney U test to determine the significance of differences in the performance of the experimental and control groups on the performance anxiety scale in the post-test.

Domains	Group	No.	Mean average	Mean total	Mean	Mann–Whitney U	z	Sig.
Somatic symptoms	Exp.	15	10.20	153.00	2.49	33.000	−3.309	0.001
	Con.	15	20.80	312.00	3.48			
Cognitive anxiety	Exp.	15	9.77	146.50	2.37	26.500	−3.582	0.000
	Con.	15	21.23	318.50	3.60			
Distractibility	Exp.	15	10.40	156.00	2.80	36.000	−3.194	0.001
	Con.	15	20.60	309.00	3.65			
Total	Exp.	15	9.07	136.00	2.56	16.000	−4.006	0.000
	Con.	15	21.93	329.00	3.58			

Table 6 shows that there is a statistically significant differences at (*α* ≤ 0.05) between the experimental and control groups in the somatic symptoms dimension. The Z-value was (Z = −3.309) at a significance level of (*p* = 0.001) in favor of the experimental group, with a mean score of (2.49) compared to (3.48) for the control group. The experimental group also had a lower mean rank (10.20 vs. 20.80). In addition; there is a statistically significant differences were also found in the anxiety dimension, with a Z-value of (Z = −3.582) at a significance level of (*p* < 0.001).

Also, the results indicate statistically significant differences in the distractibility dimension, with a Z-value of −3.194 and a significance level of *p* = 0.001, and the results show statistically significant differences in the overall score of the performance anxiety scale, with a Z-value of −4.006 and a significance level of *p* < 0.001. The mean score for the experimental group was 2.56, compared to 3.58 for the control group. The mean rank was lower (9.07 versus 21.93).

Hypothesis 2: There are no statistically significant differences at (*α*=0.05) between the mean scores of the experimental group in the post-test and follow-up on the athletes' performance anxiety scale attributable to the persistence of the training program's effect.

To verify this hypothesis, the Wilcoxon signed-rank test was used to determine the significance of the differences between the athletes' mean rank scores on the performance anxiety scale between the post-test and follow-up measurements. Table 7 presents the results.

**Table 7:** Results of the Wilcoxon signed-rank test to determine the significance of the differences between the mean scores of athletes on the performance anxiety scale between the post-test and follow-up measurements.

Dimension	Statistical Ranks (Follow Up – Post Test)	No.	Mean average	Mean total	Z	Sig.
Somatic symptoms (follow up)- Somatic symptoms (post-test)	Negative means	14	11.61	162.50	−0.755	0.450
	Positive means	9	12.61	113.50		
	Equal means	7				
	Total	30				
Cognitive anxiety (follow up)- Cognitive anxiety (post-test)	Negative means	10	6.10	61.00	−1.093	0.274
	Positive means	3	10.00	30.00		
	Equal means	17				
	Total	30				
Distractibility (follow up)- Distractibility (post-test)	Negative means	2	1.50	3.00	−1.342	0.180
	Positive means	0	.00	.00		
	Equal means	28				
	Total	30				
Total (follow up)- Total (post-test)	Negative means	16	12.19	195.00	−1.294	0.196
	Positive means	8	13.13	105.00		
	Equal means	6				
	Total	30				

Table 7 shows that there are no statistically significant differences (a = 0.05) between the athletes' scores on the performance anxiety scale in the post-test and follow-up applications in all dimensions and the overall score.

## Discussion

The findings of the present study revealed statistically significant differences between the experimental and control groups in performance anxiety, with the differences favoring the experimental group that received the stress inoculation training program for athletes. These findings suggest that the program addressed multiple dimensions of performance anxiety, including somatic anxiety, cognitive anxiety, and distractibility, reflecting a comprehensive approach to managing anxiety in sports contexts.

At the cognitive level, the reduction in cognitive anxiety among members of the experimental group may be attributed to the program's emphasis on cognitive restructuring and coping skills. These components may have helped students reinterpret performance situations as manageable challenges rather than threatening situations, thereby reducing negative expectations, fear of failure, and excessive concerns about performance (18). This finding is consistent with cognitive theories that emphasize that how an individual interprets a situation, rather than the situation itself, is a determining factor in the intensity of anxiety. Furthermore, the use of techniques such as cognitive restructuring may have reduced rumination and expectations of failure while replacing them with more realistic and positive thoughts (25).

At the physiological level, the findings indicate a decrease in the level of somatic symptoms associated with performance anxiety among members of the experimental group. This finding demonstrates the effectiveness of the training program in reducing the physiological manifestations of anxiety. Stress inoculation relies on gradual and systematic exposure to stressors combined with training in relaxation techniques, such as deep breathing and muscle relaxation. These techniques may reduce physiological arousal associated with anxiety, including muscle tension and increased heart rate. Consequently, athletes may become better able to regulate their physiological responses during competition rather than allowing these responses to interfere with their performance (17, 26).

Regarding distractibility, the significant reduction observed in the experimental group may be explained by the training provided to help students direct their attention, remain focused on the present moment, and manage both internal distractions, such as negative thoughts, and external distractions. Anxiety often consumes a significant portion of cognitive capacity, which may interfere with focusing on the primary task. The present findings are consistent with processing efficiency models, which suggest that anxiety can reduce attentional efficiency and that psychological training may help improve the effective allocation of attentional resources (26).

Overall, the findings can be interpreted in light of the program's gradual exposure to stressful situations while simultaneously equipping students with coping skills. This combination may have strengthened their ability to adapt to future stressors and enhanced their perceived control over stressful performance situations. In addition, the program may have contributed to enhancing students' self-efficacy, as mastering anxiety-management skills can provide a greater sense of control, which may reduce stress and increase self-confidence, an important factor in athletic performance

## Conclusion

This study highlights the effectiveness of a stress-proofing program as a successful intervention for reducing performance anxiety among physical education students. The results showed a clear positive effect that persisted even after the program's completion. This persistence suggests the potential for incorporating this type of program into ongoing and effective psychological interventions to address performance anxiety.

### Limitations

The limitations of this study lie in the generalizability of its findings, due to its quasi-experimental design, which may restrict complete control over all influencing variables. Furthermore, it did not address several other factors and variables associated with performance anxiety, such as prior experiences or individual differences among students, which may limit the comprehensive interpretation of the results.

### Recommendations

1. Future longitudinal studies are recommended to examine the sustainability of the effectiveness of the stress inoculation program in reducing performance anxiety over longer periods.
2. The stress inoculation program is recommended to be implemented under real-world field conditions and across different physical education contexts to further examine its effectiveness and applicability.
3. Future studies are recommended to use larger and more diverse samples to determine the generalizability of the program's findings to different groups of physical education students.
4. Future research is recommended to examine the role of individual factors and students' previous experiences in their responses to stress inoculation programs.

### Participant confidentiality

All personal information and data obtained from participants were kept strictly confidential. Identifiable details were not revealed at any stage of the research process unless explicit consent was provided by the participants.

### Equitable and respectful treatment

All participants and their responses were handled fairly and objectively, with no bias, discrimination, or manipulation during data analysis or reporting.

### Research transparency

The objectives, procedures, and findings of the study were presented clearly and accurately to ensure transparency and support the reproducibility of the research. Ethical responsibility was regarded not only as a formal requirement, but also as an essential element of scientific integrity and public confidence in research outcomes.

### Originality statement

The authors affirm that this manuscript is original, has not been published previously, and is not currently being considered for publication elsewhere. All authors have reviewed and approved the final version of the manuscript and consent to its submission to this journal.

## Data Availability

The original contributions presented in the study are included in the article/Supplementary Material, further inquiries can be directed to the corresponding authors.
